# Bursatte

**Published:** 1889-01

**Authors:** John R. Anderson

**Affiliations:** Student Chicago Veterinary College


### ART. VII.—BURSATTE.

BY JOHN R. ANDERSON,
*Student Chicago Veterinary College.*

Under this title, I wish to call attention to a disease which is at present in existence in one of the principal Southern States. As to its prevalence elsewhere I am unable to say, as my observations are confined to that particular locality, and our pathologists thus far have failed to chronicle any account of the existence of this form of the disease other than in India. The name which it bears was it seems given it by those who had its nomenclature at their disposal from the supposed influence which rainy seasons exerted in its production. The word Bursatte is derived so stated from the Hindostanee word Bursat, meaning rain. The disease itself is characterized by the formation of hard elevated patches, single or double, within the skin and cellular tissue. The chief symptoms are heat and tenderness upon pressure, and they show a tendency to enlarge and ulcerate, with a proneness to recurrence, associated with a gradual decline of the general health. The ulcers are of an extremely vegetative luxuriance and an exuberant vitality; highly vascular and becoming very malignant in their latter stages. The first appearance of the disease is marked by tumefied patches, soft at first but rapidly becoming indurated, elevated and circumscribed. The line being well marked between the morbid and normal tissue; and the skin remaining intact, presenting at this period no signs of disease beyond hyperæsthesia.

In one to six days fissures appear upon these enlargements with a yellowish brown serous exudation issuing from them, followed rapidly by ulceration, which completely divests the entire surface of the enlargement of its covering but not attacking the integument beyond the defined line. According to the most elaborate writer upon this subject there are three forms of ulcers described; each resembling the other in their primary stage, but differing materially their course of development thereafter. They are the papillated, circular medium and phagedænic. The medium he has so named as it is an intermediate stage, while the phagedænic is, as the word implies, an eating sloughing form of the disease, very destructive and exceedingly rapid in its course of development; being distinguishable from the other two forms easily, presenting as it does calcareous deposits within its substance as a result of the rapid morbid processes. This form is at present prevalent in Kansas and the northern portion of Alabama, and is the only form as he states which proves fatal although seldom. Of the three forms, there exists in this particular state only the papillated circular form, a description of which is here given as it presents itself. On completion of the removal of the integument by ulcerative process we have presented a vigorous circular ulcer the diameter of which corresponds to that of the original enlargement; not well defined at its border which is ragged; sloping from its circumference on all sides downward and inward, forming about its centre a pit-like depression, the depth of which is always proportionate to the area involved. The surface is covered with innumerable papillæ which bleed easily, and the entire ulcer has for its base a decided elevated induration which is moveable upon manipulation, seeming not to involve any tissue beyond the cellular. All of this is characteristic and taken in conjunction with the dark red jelly-like exudation that covers its surface, and is always present, is diagnostic of the disease, so decidedly that no one giving such subjects a thought can mistake it for any other ulcer presenting these conjoined features. After the maturity of an ulcer, provided it is its first appearance, repair, or rather an effort at repair, immediately follows, in which cicatrization is completed, but only remains intact for a period varying in duration from a few weeks to months when the same destructive process is again established, and the ulcer reopens but never closes the second time without medical and surgical interference, and even then only for a short while. The first manifestations of Bursatte do not bring with them any evidence of constitutional disturbance, but about the maturity of one or more ulcers a few symptoms are apparent usually, not always; such as loss of appetite, slight elevation of temperature, staring unthrifty coat, with an occasional hacking cough. All these only being transient, followed by gradual debility with its accompanying symptoms. In a few subjects which came to my notice the eyes were swollen and all the membranes infected; but this occurred only in those which presented ulcers on and about the face. When an ulcer is left at its first appearance to repair itself, the reparative process always begins at the point of first ulceration, and follows closely the destructive process. Another peculiar feature is its metastatic tendency. Should two or more tumors appear at the same time on different parts of an animal upon one or more openings simultaneously, the remaining ones disappear without doing further damage, the virus seeming to accept the first exit as a channel of escape. If the tumefactions appear upon the limbs they are to be found below the knees and hocks in every case, while those occurring elsewhere are about the face, neck, angles of the mouth, and never posterior to the anterior portion of the dorsal region. Robertson, who is the only author who gives this form of the disease any comment of importance, states that the penis, cartilaginous nictitans, vagina, internal organs, and in fact all soft structures of the body afford a site for its appearance. But that no symptoms manifest its internal existence during life beyond general debility.

On this point there is some difference, as the large external conglobate glands along their course never show signs of their involution by the disease as it occurs south of the Ohio river. It evidently occurs there in its mildest form. Again he says that an abrasion located on any part of the body occurring in a bursattic subject is very apt to assume the bursattic character. This is ever characteristically true of the affection as it appears in the locality previously referred to. It has been ascribed to a certain specific fungus, etc., but the true cause operative in its production still remains one of the unsolved pathological problems. However it is non-inoculable by direct contact to abraded muscular tissue, whatever the cause, as numerous fruitless attempts at its inoculation in this manner have proved. It cannot be transmissible by the atmosphere as a medium, as it often happens its subjects, left to mingle at will with their companions, feeding from the same mangers and water tubs for months without its transmission being effected. Those most subject to it, are hard worked, poorly fed, coarsly bred animals surrounded by most unfavorable hygenic conditions. It will probably be of interest to say that all the cases noted, save two, were in the mule, both sexes alike; although Robertson is inclined to credit this member of the equine family as well as the Jack with enjoying immunity from this strange affection. As he says, the disease is probably peculiar to the horse. This opinion is possibly accounted for from the fact that the mule is most used through this particular locality for draft purposes, and the most neglected of its species, while in the locality from which he gathers his information, the horse is used almost exclusively. It never occurs, as far as known, in any of the other domestic animals nor in the well-bred horse, kept under proper hygenic conditions. The rainy seasons, spring and autumn, may have some influence, as now thought, in its first production in an animal and prove equally influential at the same seasons in causing its aggravation in those already effected. But an animal once attacked is certainly ever after subject to recurrence at intervals of from three to five years, as proved by numerous cases. After the eradication of an ulcer on one part of the body others are found elsewhere regardless of season ; although its ravages are much modified under the influence of cold, the fact of its recurrence would point to its cancerous natnre, but when we come to sum up its periodicity, course of development, etc., as already described, this theory is at once disproven. One of the observers and speculative pathologist of this peculiar affection in his writings, the details of which will be found embodied partially on reference to Robertson's article, endeavored to prove its analogy, and in fact claimed it none other than lupus. But by comparison of the two lesions, it will be found that they are quite different affections, although admitting of the same classification. Barsattee rarely produces death of itself, and never in its papillated form, but causes such general debility, and thereby renders an animal more subject to and less capable of withstanding other diseases the most trivial of which are apt to result in death in spite of medical aid which would in all probability otherwise prove manageable. We should therefore regard the condition as a quite serious one, and we will do well also in examining for soundness to search carefully for bursattic scars and not hesitate to reject an animal presenting such evidence since the disease is certain to recur. The bursattic cicatrices are themselves very conclusive and readily detected, presenting as they do, a contracted or drawn surface with a glistening appearance resembling closely the scar of a burnt wound. In its treatment little else can be expected beyond palliation, as its true nature and causes are not at present understood. Acting at one time upon the presumption that excessive moisture was operative in producing this affection, extreme care was taken to keep the parts dry, and cauteries in the form of dry powder were used, but with less marked satisfactory result than the treatment given below. A sulphate of copper solution, say two ounces to the pint, using water as a menstrum with an addition of crystalized carbolic acid about one part to four of the solution will be found the most effectual remedy. It is to be applied daily after the ulcer has been thoroughly cauterized with the actual cautery or removed by the knife. The surgical destruction repeated, (for it will be necessary) every four to eight days, until the desired result is obtained, namely : Healthy granular action. However, care must be taken in the application of the solution as its contact with other parts will render them extremely excoriated. The internal administration of medicines seems to exert no special influence. Tonics, if desired, are admissible of course. Beyond this, with good quarters, pure air, and a liberal supply of wholesome food, little else is necessary in its treatment.

